# Effects of ethanol extract of curry leaves (*Murraya koenigii*) on HER2 and caspase-3 expression in rat model mammary carcinoma

**DOI:** 10.14202/vetworld.2021.1988-1994

**Published:** 2021-08-03

**Authors:** Siti Aisyah, Ekowati Handharyani, Nurliani Bermawie, Agus Setiyono

**Affiliations:** 1 Department of Clinic Reproduction and Pathology, Animal Biomedical Sciences Study Program, IPB University, Bogor, Indonesia; 2 Laboratory of Pathology, Faculty of Veterinary Medicine, Syiah Kuala University, Banda Aceh, Indonesia; 3 Department of Clinic Reproduction and Pathology, Division of Pathology, Faculty of Veterinary Medicine, IPB University, Bogor, Indonesia; 4 Department of Plant Genetic and Breeding, Indonesian Spices and Medicinal Crops Research Institute, Indonesian Agency for Agricultural Research and Development, Bogor, Indonesia

**Keywords:** 7,12-dimethylbenz(a)-anthracene, anti-tumor drug, apoptosis, herbal plant, human epidermal growth factor receptor 2, *Murraya koenigii*

## Abstract

**Background and Aim:**

Human epidermal growth factor receptor 2 (HER2/erbB2/neu) is a prognostic factor and biomarker for detecting mammary tumor malignancy. Leaves of curry (*Murraya koenigii*) contain alkaloid, flavonoid, and phenolic compounds that can be cytotoxic to tumor cells. Caspase-3 is an indicator of apoptosis in tumor cells. This study aimed to evaluate the effect of curry leaf extract on the expression of HER2 and caspase-3 in mammary tumor through immunohistochemical analyses.

**Materials and Methods:**

Thirty five Sprague-Dawley rats were divided into seven groups: negative control of tumor (P1), positive control of tumor (P2), tumor therapy with methotrexate (P3), and curry leaf extract doses of 300 and 400 mg/kg body weight/BW after tumor formation (P4, P5), and before tumor formation (P6, P7). Thirty rats of six groups were injected subcutaneously into the mammary glands with 7,12-dimethylbenz(α)-anthracene DMBA) twice within 2 weeks for mammary tumor formation. At the end of the treatments, the rats were euthanized, and their mammary glands were analyzed histopathologically and immunohistochemically using HER2 and caspase-3 antibodies.

**Results:**

Regarding the expression of HER2 detected in the epithelial cell membrane of the mammary gland, P2, P3, P4, and P5 revealed positive expression, P6 and P7 showed equivocal expression, while P1 showed negative expression. Regarding caspase-3 expression in the cytoplasm of epithelial cells, it was low in P1, moderate in P2, P5, P6, and P7, and high in P3 and P4. These findings suggest that DMBA injection produced mammary tumors with HER2 as a biomarker of mammary tumor, and high caspase-3 expression in P4 was the effect of curry leaves extract.

**Conclusion:**

The extract of curry leaves at a dose of 300 mg/kg BW with preventive and curative effects can potentially be used as an anti-tumor agent, which effectively induces the apoptosis of tumor cells.

## Introduction

Breast cancer remains the most prevalent cancer type in Indonesia, accounting for 16.7% of total tumors and having a mortality rate of 17% [1]. One approach to lowering the mortality due to mammary tumors is the early detection of mammary tumor malignancy. However, molecular analysis of tumor tissue only by pathological examination is inadequate for this purpose. An immunohistochemical examination is required to determine the developmental stage and receptor status, as well as to evaluate the efficacy of a given treatment [2]. Treatment strategies in malignant tumors were evaluated based on the correlation of clinical results with diagnostic pathology [3]. Several biomarkers of breast tumors with prognostic or predictive value have been established, including estrogen receptor, progesterone receptor, and human epidermal growth factor receptor 2 (HER2) [4,5]. HER2 receptor can be used as an indicator for determining the malignancy of mammary tumors and suitable therapy. HER2/erbB2/neu is a transmembrane protein receptor in the epidermal growth factor receptor group. HER2 has tyrosine kinase activity, in which receptor dimerization is produced during autophosphorylation of tyrosine residues within the cytoplasm domain receptor and initiates various signaling pathways that cause cell proliferation and tumorigenesis [5-7]. The overexpression of HER2 was found in 20-30% of mammary tumor cases. It is related to disease aggressiveness, high disease recurrence, and increased mortality [8-10].

Preventing tumors from developing to a later stage or lowering tumor malignancy can be achieved by evaluating the effectiveness of a given therapy. Evaluation of the effectiveness of therapy can be done by examining apoptosis. Apoptosis is a programmed cell death. Disturbed apoptosis is one of the characteristics of tumor development. Caspase-3 is the main initiator of the apoptotic cascade in tumor cells and is an important biomarker of apoptosis. The development of mammary tumors is closely related to the expression of caspase-3. Caspase-3 is part of the cysteine-aspartate protease acid (caspase) family, which plays a role in the apoptotic signaling pathway and regulates apoptosis [11]. The inhibition of apoptosis in tumor cells can increase bad prognosis. Various researchers have used apoptosis as the main target in the search for anti-tumor drugs [12-14]. Secondary metabolites in plants have high potential as anti-tumor drugs because it can cause cytotoxic and apoptosis in tumor cells, so that growth can be inhibited. Secondary metabolites with anti-tumor activity include alkaloids, flavonoids, and terpenoids [15].

One of the herbal plants containing these secondary metabolites is curry (*Murraya koenigii*), specifically, its leaves. In various studies, curry leaves have been proven to have anti-tumor effects both *in vitro* [16-19] and *in vivo* [20-22]. Studies indicated that curry leaves’ alkaloid compounds could induce apoptosis in the mammary cancer cells MCF7, HeLa, P388, and HL-60 [23,24], as well as colon cancer cells HT-29 [25]. It was also described that flavonoid and phenolic compounds of curry leaves can be effective at preventing colon cancer *in vitro* [26].

However, *in vivo* research on curry leaves is still limited. This study was established to evaluate the effect of ethanol extract of curry leaves on the expression of HER2 and caspase-3 in mammary gland tumors in Sprague-Dawley (SD) rats through immunohistochemical analyses. HER2 expression was analyzed to reflect mammary tumor malignancy after the administration of 7,12-dimethylbenz(a)-anthracene (DMBA), whereas the expression of caspase-3 was analyzed to show apoptosis of mammary tumor cells.

## Materials and Methods

### Ethical approval

The Ethical Committee has approved this research of Faculty of Veterinary Medicine, IPB University, Bogor, under certificate No. 094/KEH/SKE/VIII/2018.

### Study period and location

The study was conducted from August 2018 to August 2019 at the Laboratory Animal Management Unit and Laboratory of Pathology, Faculty of Veterinary Medicine, IPB University, Bogor, Indonesia.

### Curry leaf preparation

A certified botanist performed taxonomic determination at the Herbarium Bogoriense LIPI Bogor. Curry leaves were obtained from around the Faculty of Veterinary Medicine, IPB University, Bogor. The leaves were aromatic, bright green, and shiny. The leaves were dried, ground, and sieved through a mesh 60 sieve. The dried leaves were then extracted with 70% ethanol using a maceration method. Curry leaf extract was then concentrated by a rotary evaporator at 40°C and 50 rpm until a thick extract was obtained (Pharmacy Laboratory, Faculty of Veterinary Medicine, IPB University).

### Experimental design

Female SD rats aged 3-4 months were obtained from the laboratory animal unit (UPHL), Faculty of Veterinary Medicine, IPB University. The rats were maintained in a well-ventilated room with a 12-h light/dark cycle and feed and water were supplied *ad libitum*. Mammary tumor was induced using (DMBA; Sigma) at 20 mg/kg body weight(BW) dissolved in 1 ml of olive oil injected subcutaneously into rat mammary glands twice with an interval of 7 days. Thirty-five female SD rats were divided into seven groups (each group consisting of five rats). P1 was the normal group, which was only given a placebo (2 ml of distilled water for each experimental animal). P2 to P7 were the tumor treatment groups, treated with DMBA. P2 was the tumor control group, given distilled water. P3 was the control medicine group, given methotrexate (MTX) at 0.125 mg/kg BW after tumor formation. The curative groups (P4 and P5) were given curry leaf ethanol extract (CLEE) at 300 or 400 mg/kg BW after the tumor formed, while the preventive groups (P6 and P7) were given CLEE at 300 and 400 mg/kg BW along with DMBA injection before the tumor formed. The extract and MTX were given to the rats orally for 30 days. Tumor palpation of the mammary gland was conducted after the last DMBA injection.

### Immunohistochemical staining and histopathological examination

At the end of the treatment, all rats were euthanized and the mammary tumors were fixed in 10% neutral buffered formalin. Paraffin blocks were made and cut into 5 μm slices for each rat. Three consecutive slices were stained immunohistochemically. Slides were incubated in antigen retrieved (pH 6.0), for 15 min at 95°C, cooled at 27ºC (room temperature/RT) (for caspase-3 antibody), followed by washing with PBS. Slides were then incubated with endogenous activity blocking H_2_O_2_ 3% (Biocare Medical, California, USA) (HER2 and caspase-3 antibody) for 30 min at RT. Next, slides were incubated with primary antibody against HER2 (1:100; mouse monoclonal [3B5] antibody to ErbB2, ab16901; Abcam, Cambridge, UK) and caspase-3 (1:250; rabbit polyclonal antibody to caspase-3, ab4051; Abcam) at 4°C overnight. Slides were incubated with a secondary antibody (Trekkit Universal Link; Biocare Medical) for 30 min at RT and were then incubated with Trek Avidin-HRP Label (Biocare Medical) for 30 min at RT. Following rinsing with PBS, visualization was performed using the peroxidase substrate 3,3-diaminobenzidine (Biocare Medical) as the chromogen. Then, counterstaining with hematoxylin was carried out, followed by mounting. Apart from the palpation method, the presence of a tumor mass was also confirmed through hematoxylin-eosin and Masson’s trichrome staining.

### Statistical analysis

Data were analyzed semi-quantitatively based on the immune reaction using a light microscope. A score was assigned based on the average value of each treatment group. Observations on each slide were carried out based on ten fields of view at 20× magnification. HER2 expression was observed based on the staining intensity of the epithelial cell membrane in mammary glands with the following categories: HER2-negative (−): cell membrane unstained or ≤10% incompletely stained tumor cell membrane, (+): >10% tumor cell membrane incompletely stained, HER2-equivocal (++): >10% tumor cell membrane incompletely stained or weakly stained or ≤10% tumor cell membrane completely stained, and HER2-positive (+++): >10% tumor cell membrane homogeneously stained [27]. Caspase-3 expression was observed based on staining intensity in mammary gland cell cytoplasm with the categories of low (+), moderate (++), and high (+++).

## Results

**Figure-1:**
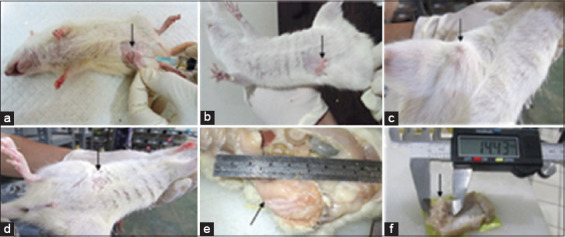
Mammary tumors in Sprague–Dawley rats. (a) Injection of 7,12-dimethylbenz(α)-anthracene (DMBA) into the subcutaneous of the right mammary gland, (b) 7 days after the initial injection of DMBA, (c) Tumor nodules were visible on the skin surface, (d) Tumor nodules were not visible on the surface of the skin, (e and f) The tumor adhered to the skin and abdominal wall.

The results showed that breast tumor nodule formation could be detected by palpation in about 40-60% of the rats at 14 days after DMBA injection, while on the 21^st^ day, tumor nodules had formed in 100% of the injected rats. Figure-1 shows the clinical features of the mammary glands on the 7^th^ day after the initial injection of DMBA. The mammary glands became inflamed, red, and had nodules with a soft consistency. Palpation of the mammary gland tumor nodules as performed on day 14 showed a spongy to hard consistency. In terms of the consistency of the tumors, they were single solid masses adhering to the skin and abdominal wall. The shapes of the tumors varied, such as being round, oval, and flattened. Some of the tumor nodules were visible on the surface of the skin, while others were not. Tumors that were not visible on the surface of the skin indicated tumor progression into the abdomen.

**Figure-2:**
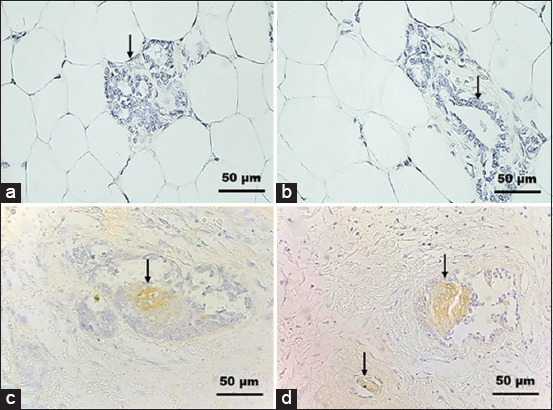
Expression of human epidermal growth factor receptor 2 (HER) on the membrane of the epithelial cells of the mammary gland (arrow). (a and b) The mammary gland a negative control in the P1 group. (c) Equivocal expression (++) in the P6 group. (d) Positive expression (+++) in the P4 group. HER2 immunostaining, hematoxylin counterstain.

The results of HER2 and caspase-3 expression in SD rats as revealed by IHC are shown in Table-1. HER2 protein was homogeneously expressed on the epithelial cell membrane of mammary glands. Evaluation of HER2 protein was indicated by the stained cell membrane. Membrane cells were light brown stained incompletely (++/equivocal) and light/dark brown stained completely (+++/positive) (Figure-2). HER2 overexpression in rat mammary tumors was found by epithelial cell proliferation in mammary gland duct and epithelial cells located between polymorphonuclear (PMN) cells.

**Table 1:** Expression of HER2 and caspase-3 in Sprague–Dawley rats.

Groups	Treatment	HER2 expression	Caspase-3 expression
P1	NC	−	+
P2	TC	+++	++
P3	TT+MTX	+++	+++
P4	TTC+CLEE 300 mg/kg BW	+++	+++
P5	TTC+CLEE 400 mg/kg BW	+++	++
P6	TTP+CLEE 300 mg/kg BW	++	++
P7	TTP+CLEE 400 mg/kg BW	++	++

HER2=Human epidermal growth factor receptor 2, NC=Normal control, TC=Tumor control, TT=Tumor treatment, MTX=Methotrexate, CLEE=Curry leaves ethanol extract, C=Curative, P=Preventive

The HER2 overexpression indicated the malignancy of mammary gland tumors. Based on HER2 overexpression, P2, P3, P4, and P5 groups were diagnosed as having malignant tumors due to mammary carcinogenesis induced by the two subcutaneous injections of DMBA. DMBA proved to be highly carcinogenic to the rats and induced malignancy in the P2, P3, P4, and P5 groups. Meanwhile, in the P6 and P7 groups, equivocal HER2 expression was observed. It was thought that secondary metabolic compounds in CLEE may inhibit tumor cell development. This was proven by the finding that, when CLEE was stopped, normal cells began to proliferate again into tumor cells.

**Figure-3:**
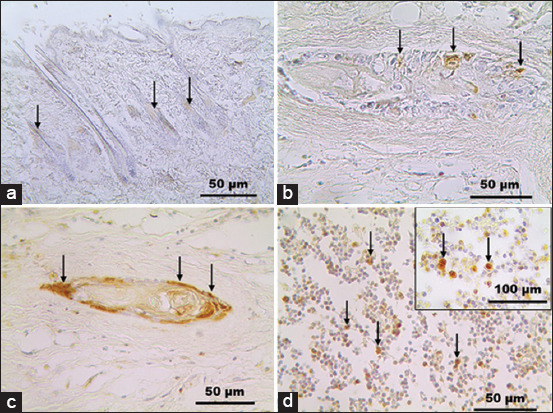
Expression of caspase-3 (arrow). (a) The hair follicles of the skin of Sprague–Dawley rats as negative control are low immunoreactivities for caspase-3 in the P1 group. (b) Mammary gland epithelial cells are overexpressed of caspase-3 in the P2 group. (c) Duct epithelial cells (d) Epithelial cells were outside the duct of the mammary gland in the P4 group. Caspase-3 immunostaining, hematoxylin counterstain.

Caspase-3 expression showed brown-stained cell cytoplasm, which indicated the presence of apoptotic cells (Figure-3). Caspase-3 expression could be found in the tumor control group and treatment groups given MTX and CLEE at dosages of 300 and 400 mg/kg BW. This marker was used to evaluate the response to drug and extract treatments. In the control P1 group, apoptotic cells were detected in skin epidermis, hair follicles, and sebaceous glands in the mammary gland skin (+). Caspase-3 overexpression was moderate (++) in groups P2, P5, P6, and P7. Meanwhile, there was overexpression (+++) of caspase-3 in groups P3 and P4. Overexpression was found in mammary gland duct epithelial cells, epithelial cells outside the duct, fibrous cells, and PMN cells.

MTX (P3) and CLEE at a 300 mg/kg BW dosage (P4) showed similarly high apoptotic activity in HER2-positive mammary tumor cells. A high number of apoptotic cells were obtained with CLEE at a dose of 300 mg/kg BW, showing its effectiveness against malignant tumor cells. Therefore, it can be used as an anti-mammary tumor candidate.

## Discussion

In this study, DMBA injection showed that the majority of mammary tumors were HER2-positive. The American Society of Clinical Oncology and the College of American Pathologists recommend that patients with mammary tumor can be characterized as being in the early or advanced stage based on HER2 status (negative, equivocal, or positive), using one or more HER2 tests. HER2 positivity in an IHC test is defined when more than 10% of adjacent tumor cells homogeneously express HER2 in the cell membrane [4]. The categories are defined as follows: unstained or weakly and incompletely stained surrounding cells (0/+), surrounding cell membrane weakly stained or moderately stained at >10% or membrane strongly stained in ≤10% of tumor cells (++), and cell membrane strongly stained in >10% of cells (+++) [28]. Overexpression of HER2 could be found in 15-20% of invasive ductal carcinoma mammary tumors with intense brown color on the cell membrane from weakly to moderately stained in >10% of tumor cells [29].

The overexpression of HER2 is a poor prognostic factor associated with inadequate differentiation, high cell proliferation, metastasis to lymph nodes, and often resistance to specific chemotherapy. HER2 overexpression is also associated with a higher risk of recurrence compared with that in HER2-negative mammary tumor [30]. The HER group plays an important role in the signaling pathway controlling cell growth and differentiation. HER2 overexpression is necessary for cancerous transformation and tumorigenesis [31]. HER2 activates the phosphatidylinositol-3 kinase-AKT-NF-kB pathway to stimulate the mitogenic cyclic D1/CdK4-Rb-E2F pathway. HER2 overexpression also stimulates the autonomous tumor cell suppressor DmP1-Arf-P53 pathway to suppress the oncogenic signal, which results in the appearance of tumor cells [8]. Activity of the HER2 constitutive homodimer occurs due to HER2 overexpression on the cell surface. This makes cell growth unregulated and leads to oncogenic transformation. HER2 overexpression also contributes to malignant tumor growth through the recruitment of other HER receptors, especially HER3. HER3 has been shown to cause an increase in phosphotyrosine in HER2-positive tumors [31,32]. In the study, CLEE at a dose of 300 mg/kg BW given preventively showed the potency of CLEE to suppress tumor cell growth with HER2 level (++),while CLEE given curatively still showed HER2 level (+++). However, the potency of CLEE for a curative effect was showed in apoptosis with caspase-3 level (+++), similar to that of MTX therapy.

Apoptotic cells can commonly be found in normal tissue, but at a low level. Caspase-3 expression is commonly found in cells in the body. In normal skin, caspase-3 expression-positive cells can be found in epidermal cells, namely, hair follicle epithelial cells, and sebaceous gland epithelial cells [33]. The characteristics of Caspase-3, as shown by IHC showed homogeneous brown positivity in the cell cytoplasm [34]. This research showed that high caspase-3 overexpression was found in the MTX-administered group (P3) and curative group (P4), which was given CLEE at a dosage of 300 mg/kg BW. High caspase-3 overexpression indicated that the mammary gland tumor cells underwent apoptosis, shrinking the tumor. This is in accordance with previous research showing that the tumor size was smaller in an MTX-administered group (P3) and a curative group (P4) given CLEE at 300 mg/kg BW. Palpation of the mammary glands revealed tumor nodules with a firm consistency. Histopathological imaging revealed neoplastic cells, fibroblast cell proliferation, glandular duct proliferation, and glandular epithelial cell proliferation. The tumor size in group P2 was greater than that in groups P3, P4, P5, P6, and P7. Meanwhile, the smallest tumor size was seen in the P3 and P4 groups [22].

The obtained results indicated that secondary metabolites contained in CLEE, especially alkaloid, terpenoid, and flavonoid compounds, may act as anti-tumor drugs. This is in line with the previous findings that compounds could suppress cell growth through DNA damage and apoptosis. Apoptosis could be detected through caspase-3 protein was one of the effector caspases helped by initiator caspase, which is very important in the apoptotic pathway [35-37]. Curry leaves are rich in bioactive compounds such as alkaloids and flavonoids, which are proven to improve caspase-3 activity by increasing mammary tumor cell apoptosis in nude mice and human mammary cancer cells (MDA-MB-231) [38]. Alkaloid compounds (murrayazoline and o-methylmurrayamine) were shown to activate the caspase-3 protein, which regulated the induction of apoptosis in colon cancer cells (DLD-1) [36]. Compounds in curry leaves have also been reported to selectively inhibit cell proliferation and induce cell apoptosis in lung cancer cells A549. These compounds can increase the regulation of caspase-3, -7, and -9, and release cytochrome c into the cytosol. This supports the involvement of mitochondria in the apoptotic process caused by alkaloid compounds in A549 cells [39].

In this research, the injection of DMBA led to the formation of malignant tumors (cancer) in mammary glands of rats with a HER2-positive status. The P4 group with HER2 positivity given CLEE at 300 mg/kg BW showed high overexpression of caspase-3. This is thought to be due to the high activity of secondary metabolic compounds in CLEE, which can cause apoptosis in tumor cells. Meanwhile, the activity of these CLEE compounds in the P5, P6, and P7 groups with a HER2-equivocal status led to moderate overexpression of caspase-3. Curry leaf extract can reduce cell viability and proliferation of MCF-7 and MDM-MB-231 breast cancer cells, while having no effect on normal cells [21]. Curry leaf extract inhibits the formation of mammary tumor, reducing mitotic cells and cytotoxicity [20].

## Conclusion

Subcutaneous DMBA injection into the mammary glands can rapidly induce the formation of a mammary tumor within 14-21 days. The majority of mammary tumors formed were HER2-positive. CLEE at a dose of 300 mg/kg BW in both curative and preventive administration had anti-tumor potency, which could effectively cause caspase-3 overexpression, associated with the apoptosis of tumor cells.

## Authors’ Contributions

SA and AS: Designed the study. SA: Carried out the preparation of the sample. SA, EH, and NB: Performed data collection, statistical analysis, data interpretation, and manuscript writing. SA, NB, and AS: Involved in the monitoring of research and manuscript editing. All authors have read and approved the final manuscript.
